# Expiratory Musculature Targeted Resistance Training Modulates Neural Connections During Swallowing Tasks: Preliminary fMRI Evidence

**DOI:** 10.1155/np/2075288

**Published:** 2025-08-06

**Authors:** Rahul Krishnamurthy, Douglas H. Schultz, Yingying Wang, Steven M. Barlow, Angela M. Dietsch

**Affiliations:** ^1^Department of Neurosurgery, University of Nebraska Medical Center, Omaha, Nebraska, USA; ^2^Department of Special Education and Communication Disorders, University of Nebraska–Lincoln, Lincoln, Nebraska, USA; ^3^Center for Brain, Biology, and Behavior, University of Nebraska–Lincoln, Lincoln, Nebraska, USA; ^4^Department of Psychology, University of Nebraska–Lincoln, Lincoln, Nebraska, USA; ^5^Department of Biological Systems Engineering, University of Nebraska–Lincoln, Lincoln, Nebraska, USA

**Keywords:** deglutition, dysphagia, EMST, sensorimotor plasticity

## Abstract

**Purpose:** Strength gains and synergistic muscle group activations due to expiratory muscle strength training (EMST) lead to beneficial changes in several upper aerodigestive functions, including swallowing; however, there may be a potential top–down influence through neuroplasticity. The current study investigated changes in brain activation patterns during swallowing tasks before and after 4 weeks of EMST.

**Methods:** Five right-handed, healthy young adult men aged 19–35 (mean = 28.8, SD = 2.68) participated in 4 weeks of EMST. All participants performed a swallow task, and blood–oxygen level-dependent (BOLD) responses were obtained at baseline and post-training conditions using functional magnetic resonance imaging (fMRI).

**Results:** We observed a significant increase in activation across 12 regions, including the left primary somatosensory cortex (S1), left primary motor cortex (M1), bilateral supplementary motor areas (SMAs), bilateral cerebellum, right middle frontal gyrus, insula, anterior cingulate, and thalamus, following 4 weeks of EMST. While activations in several regions implicated with swallowing were expected, we also observed strong activation in areas associated with motor learning and cognitive functions.

**Conclusion:** Our study's results provide preliminary evidence that EMST can modulate neural networks associated with swallowing. We speculate that enhanced sensorimotor excitability and cortical representation, motor learning, and improved cognitive-sensorimotor integration contribute to EMST's multidomain benefits. Furthermore, our findings suggest that EMST may offer potential cognitive and neuroprotective benefits beyond improving upper aerodigestive functions.

## 1. Introduction

Resistance (strength) training of the expiratory musculature, such as expiratory muscle strength training (EMST), improves the functional strength of the abdominal and intercostal muscles and synergistically activates the upper aerodigestive tract. These strength gains and synergistic muscle group activations lead to beneficial changes in several upper aerodigestive functions, including swallowing through multiple peripheral (physiological) mechanisms [1–8]. While the multidomain benefits of EMST are reported to be likely driven by peripheral processes, mainly via stimulation of the respiratory and upper aerodigestive muscles, a top–down contribution through neuroplasticity could also significantly contribute to the benefits observed across upper aerodigestive functions, such as swallowing [9, 10].

In their seminal paper, Sapienza and Wheeler-Hegland [8] speculate that EMST may enhance the sensory and motor representation and excitability of brain areas associated with the muscles involved, and these benefits are thought to transfer across skilled tasks, such as swallowing. The exercise science literature also extensively debates the notion that strength training can influence skilled behaviors through neuroplasticity [11–16]. The response to a strengthening stimulus is training-specific, that is, the strength gains are most pronounced in movements similar to those practised [14–16]. In their review, Caroll et al. [11] acknowledge the training-specific nature of strengthening regimens and further argue that the principle of training specificity does not entirely rule out the potential for broader neuroplastic benefits through motor learning. Moreover, studies have shown that resistance training results in adaptations at both supraspinal (cortical) and corticospinal levels. A study by Caroll et al. [12] found that resistance training involving sustained maximal force production through simple repetitive movements reorganized the input–output properties of the cortico-spinal pathway. Another study by Selvanayagam et al. [15] demonstrated that resistance training showed neural adaptations similar to motor learning, and resistance training might be analogous to the process of learning to optimize muscle activation patterns to enhance force production. Additionally, a recent review by Tallent et al. [17] concludes that motor skill and resistance training at the cortical level seem to have similar neuroplastic adaptations with a release of intra-cortical inhibition and increased cortico-spinal excitability.

Our previous studies partially support this idea, demonstrating that 4 weeks of EMST not only strengthens the expiratory musculature but also induces molecular and neuroplastic adaptations [9, 18]. In our previous experiments, healthy men completed five sets of five repetitions of EMST per day, 5 days per week, for 4 weeks at 75% of their maximum expiratory pressure (MEP; [19]). Behavioral, biochemical, and neuroimaging data were collected at baseline and after the 4 weeks of intervention. We observed a significant increase in MEP and circulating serum levels of brain-derived neurotrophic factors (BDNFs). Furthermore, task-based functional magnetic resonance imaging (fMRI) revealed that after training, EMST increased the brain's ability to differentiate between swallowing tasks and rest. This effect was observed in eight key areas: the bilateral primary motor (M1) and somatosensory cortices (S1), bilateral insula, supplementary motor area (SMA), and cerebellum [18]. These activation patterns suggest that EMST induces plasticity within regions associated with motor control adaptation (M1), learning (SMA), and sensory processing (S1), thereby providing preliminary evidence for the neuroplastic potential of EMST. However, the task-specific influences of EMST on swallowing-related neural activity remain largely unexplored.

The current research note addresses this gap by investigating how the neural activity related to swallowing changes following EMST. Based on earlier reports [11, 12, 15], we speculate that sensorimotor adaptations from resistance training may extend beyond strength gains, potentially enhancing neural control and execution of skilled behaviors, such as swallowing, through neuroplasticity. We hypothesize that, for the swallowing task, EMST will lead to increased activations in well-established regions implicated in swallowing, such as the S1, M1, insula, and cerebellum [20–25].

## 2. Methods

### 2.1. Participants and Training

Five right-handed, healthy young adult men aged 19–35 (mean = 28.8, SD = 2.68) were recruited from the community. All participants had normal lung volumes and capacities, a healthy BMI (mean = 21.86, SD = 1.06), and no self-report history of smoking or pulmonary disorders. The exercise training was carried out using the EMST-150 device (aspire respiratory products) and followed the protocol proposed by Sapienza [19]. The procedure involved measuring the participant's baseline MEP using custom-developed instrumentation reported in the Dietsch et al. [26] study and setting the initial resistance level to 75%_MEP_. In each session, our participants exhaled against the set resistance level under a controlled dosage of five sets of five breaths x 5 days/week over 4 weeks. At the end of every week, we assessed their expiratory strength gains, that is, MEP, and the therapeutic resistive load was adjusted progressively.

### 2.2. fMRI Acquisition Parameters and Timeline

For each participant, high-resolution T1 anatomical, task-based fMRI, diffusion-weighted images (DWIs) and field map scans were acquired on a Siemens 3T Magnetom Skyra. The technical specifications of T1 anatomical were as follows: TR = 2200 ms, TE = 3.37 ms, flip angle = 7°, FOV = 256 mm, slice thickness = 1 mm, and field map sequences were as follows: TR = 523 ms, TE 1 = 4.92 ms, TE 2 = 7.38 ms flip angle = 60°, FOV = 220 mm, slice thickness = 2.5 mm. The imaging parameters for DWI were as follows: flip angle/TE/TR/TA/FOV = 90°/88/8320 ms/5:59 min/256 × 256 mm; *b* = 1000 s/mm^2^. The baseline scans were obtained 1 day before the beginning of the training, and the post-training scans were obtained within 3 days of completing the training.

### 2.3. fMRI Task Stimuli and Blood–Oxygen Level-Dependent (BOLD) Responses

We used a block design with a swallow task and rest period. A single functional run comprised 10 saliva swallow blocks and 10 alternating rest periods, 10 s each in duration, along with a jittered intertrial interval (3, 4, or 5 s) presented in a pseudorandom order through LabChart. All participants completed three functional runs in each neuroimaging session, totaling 12 min (3 runs × 4 min). Functional images were acquired using the BOLD contrast, which measures neural activity indirectly through changes in deoxygenated hemoglobin concentration. When a brain region becomes active during a task like swallowing, the local demand for oxygen increases, increasing blood flow. This supply of oxygenated blood exceeds the brain's consumption, which results in a relative decrease in deoxygenated hemoglobin, thus increasing the BOLD signal. This signal serves as a proxy for neural activation, allowing us to assess brain regions involved in swallowing. Given our study's aim to investigate the effects of EMST on neural control of swallowing, comparing BOLD responses during the swallowing task and rest periods allowed us to assess specific neuroplastic changes within swallowing-implicated regions as a result of training.

### 2.4. fMRI Preprocessing and Analyses

The fMRI data were processed using AFNI [27] and FreeSurfer pipelines [28, 29]. Details of preprocessing are described in our previous study [18]. For task-related analysis, a general linear model was applied using a “BLOCK” basis function (20 s duration, peak amplitude = 1) to model the hemodynamic responses. These regressors were incorporated into the GLM to identify brain activation patterns for swallow tasks across baseline and post-training conditions. Finally, multivariate modeling was performed using AFNI's 3dMVM program [30]. Spatial smoothness was estimated using the 3dFWHMx function, and autocorrelation values were calculated for each participant (mean: 0.746, 2.138, and 6.549). Cluster-based correction for multiple comparisons with a bi-sided NN = 2 option was applied [31], with a minimum cluster size of 18 contiguous voxels and a voxel-wise *p* value of 0.005, resulting in a corrected alpha of *p*  < 0.05. Beta weights associated with the changes in neural regions associated with the swallow task across baseline and post-training conditions were extracted for further analysis.

## 3. Results

**Figure 1:**
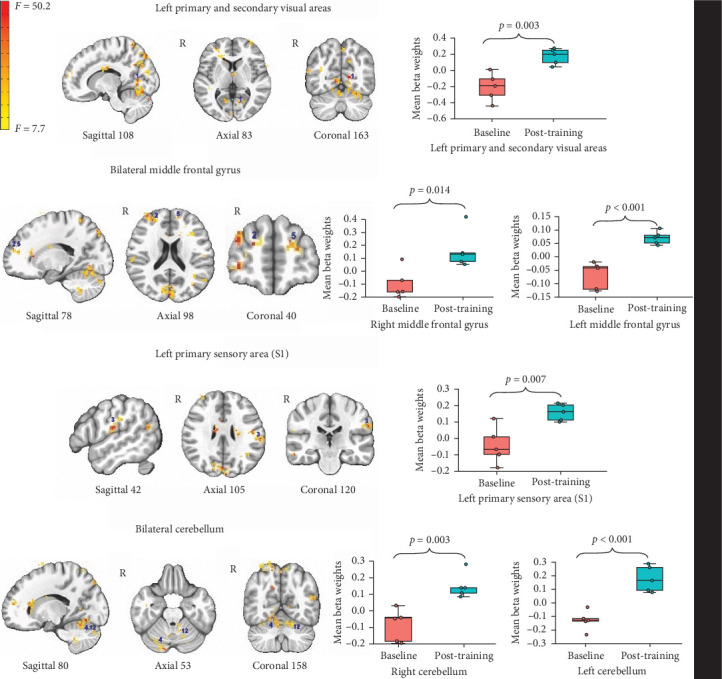
Areas of activation associated with swallowing task and changes in its beta weights across baseline and post training. *Note:* Whiskers represent standard error; the color bar reflects *F*-values. The reported *p* values reflect the mean of voxel-wise *p* values within each significant cluster.

**Figure 2:**
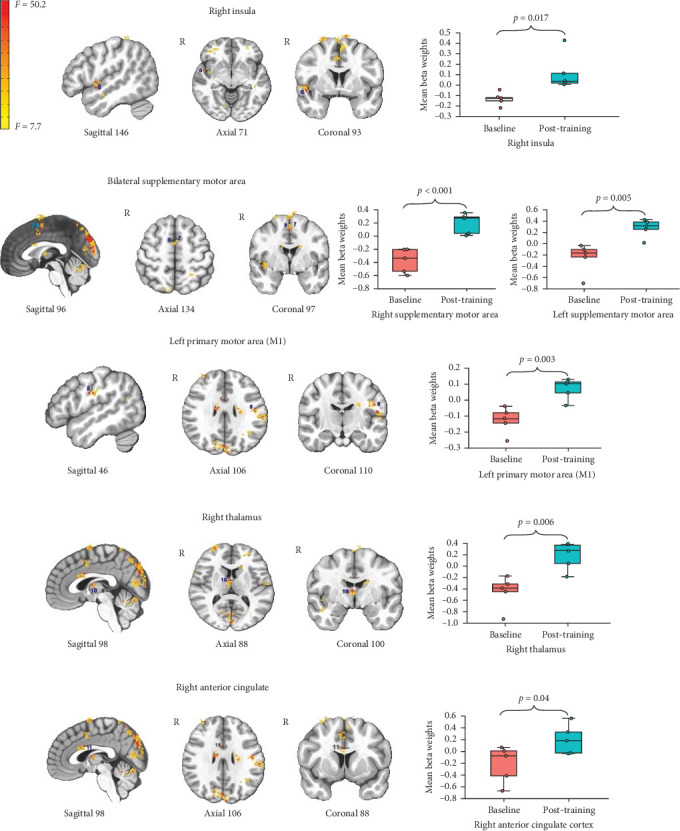
Areas of activation associated with swallowing task and changes in its beta weights across baseline and post training. *Note:* Whiskers represent standard error; the color bar reflects *F*-values. The reported *p* values reflect the mean of voxel-wise *p* values within each significant cluster.

For the effect of EMST on BOLD activity during the swallowing task (vs. rest), we observed significant activation in 12 regions, including the left S1, left M1, bilateral SMA, bilateral cerebellum, bilateral middle frontal gyrus, right insula, right anterior cingulate, and right thalamus. The pattern of this effect was such that there was greater activation post-training compared to the baseline for all 12 regions. Figures 1 and 2 show the 12 regions and the changes in their beta weights.  Table 1 shows the coordinates, cluster size, and effect descriptors for the 12 neural regions. Activations in the visual cortices were as expected given the visual presentation of the task stimuli.

## 4. Discussion

Previous studies have established that EMST offers significant benefits across several upper aerodigestive functions, including voice, cough, and swallowing [5–8, 32]. These functional improvements are underpinned by strength gains that likely transfer across multiple upper aerodigestive functions, as well as by enhanced intermuscular coordination (synergistic activation) and neural adaptations in motor control, learning, and sensory processing regions [10, 18]. Despite these well-documented functional benefits, the top–down mechanisms of EMST and its modulatory effects on swallowing-related neural activity remain underexplored.

Our previous studies demonstrated that EMST not only strengthens expiratory musculature but also induces molecular and neuroplastic changes [9, 18]. Specifically, we observed quantitative increases in circulating serum levels of BDNF (*p*=0.006) and insulin-like growth factor 1 (IGF-1; *p*=0.951) following 4 weeks of EMST. These molecular markers are well-established mediators of neuroplasticity and have been implicated in synaptic plasticity, neurogenesis, and motor learning [33–37]. These changes were accompanied by increases in MEP and enhanced activation across eight brain regions, including areas associated with sensorimotor control and learning, supporting the notion that EMST promotes functionally relevant neuroplastic and molecular adaptations. In the current study, we aimed to investigate the task-specific effects of EMST on swallowing-related neural activity.

The results of the current study demonstrate that EMST induces significant changes in neural activation patterns associated with swallowing and facilitates the modulation of swallowing-related neural networks. As hypothesized, we observed significantly increased activation in the left S1, left M1, bilateral SMA, and bilateral cerebellum, as well as in the bilateral middle frontal gyrus, right insula, anterior cingulate, and right thalamus during the swallowing task following EMST. These activations align with the previous studies that have implicated similar gray matter regions in the neural control of swallowing [20–25]. Furthermore, the bilateral nature of the observed activations is consistent with the existing literature [22, 23].

In addition to bilateral activations, hemispherical dominance for swallowing has also been reported and discussed in the literature [22, 38, 39]. The observed lateralization, with greater activation in the right cerebellum, may reflect its functional specialization in coordinating midline and axial musculature involved in expiratory effort [40] and swallowing [41, 42]. Notably, the right cerebellum projects via the dentate nucleus to the left primary motor cortex, potentially aligning with dominant hemispheric control of volitional respiratory functions [40, 43]. In parallel, activation in the right thalamus is consistent with this cerebello–thalamo–cortical projection pathway and may reflect lateralized involvement in motor planning and control during EMST. We speculate that this asymmetry may reflect broader hemispheric differences in sensorimotor integration or increased cognitive-motor demands processed predominantly through right-sided cerebello–thalamic circuits. Future research is warranted to better understand the functional significance of these asymmetries and how they might be leveraged in the design of targeted swallowing therapies.

EMST has been shown to improve swallowing safety and efficiency by activating the suprahyoid musculature, which promotes greater movement of the hyolaryngeal complex and facilitates the increased opening of the upper esophageal sphincter [7, 8]. Beyond peripheral synergistic activations, two top–down mechanisms are thought to contribute to the improvements in swallowing function resulting from EMST: (1) enhanced sensorimotor excitability and representation, and (2) motor learning benefits [10, 18]. In addition to these two mechanisms, we speculate that improved cognitive-sensorimotor integration may also contribute to the observed improvements in swallowing function.

Sensorimotor excitability and representation refer to the brain's ability to increase sensitivity and responsiveness to sensory input and/or motor commands, as well as fine-tuning the mapping of these functions within the cortex. Our findings show increased activation in the S1 and M1 areas, traditionally associated with sensory and motor processing. These changes suggest that EMST may improve the brain's ability to fine-tune the mapping and responsiveness of sensory and motor functions, contributing to improved coordination of muscle activity. As a result, this heightened cortical activation may lead to more precise and efficient muscle control during tasks that require skilled sequencing, such as swallowing [10]. However, further investigation is needed to understand the broader implications of these changes, particularly in terms of how they affect sensorimotor networks within the brain. Exercise science literature has shown that resistance training can modulate a range of well-known neural networks [44–46], and future research should explore changes in intrinsic brain activity (resting-state functional connectivity) to examine how EMST modulates the connectivity between sensorimotor networks and other known motor networks, such as the fronto–parietal network.

Secondly, we speculate that EMST involves some degree of novel motor skill learning, and the neural benefits associated with motor learning may potentially transfer to drive benefits across upper aerodigestive functions, including swallowing. This assumption is further supported by strong bilateral activations in SMA and cerebellum, key regions involved in motor learning, planning, coordination, and fine-tuning of motor output. These activations suggest that EMST may not only engage the sensorimotor networks (M1, S1) but also tap into broader neural processes related to motor planning, coordination, and adaptation (bilateral SMA and cerebellum). Moreover, these observations reinforce that EMST is not a simple strength exercise but somewhat mimics skill-based intervention involving dynamic learning processes.

Motor learning resulting from a resistance training regime occurs partly due to changes in the strength of connections between neurons in supraspinal motor centers [47]. Furthermore, the improved connectivity within motor pathways due to repeated practice may not only enhance the specific motor task trained but also have broader implications for movements requiring similar neuromotor control and/or coordination [11, 16]. We believe that similar motor learning benefits occur during EMST; furthermore, it may induce changes in the neural pathways that control muscle activation and coordination. These changes occur through increased synaptic efficacy within motor circuits, refining the brain's control over the muscles involved in the task [11]. Collectively, alterations in synaptic efficacy within these pathways could impact muscle activation during other tasks, such as swallowing, that are thought to engage overlapping circuits.

Exercise (both skill and strength) training has been well-documented to alter gray matter and white matter volume in areas of the brain associated with motor control, learning, and memory [48–51]. For instance, long-term motor learning, such as playing an instrument, learning novel speech motor sequences, and engaging in complex motor tasks, has been shown to lead to structural changes in the brain, particularly in areas like the motor cortex, cerebellum, and hippocampus [48–50, 52]. These structural changes are thought to underlie the neuroprotective benefits of exercises (strength or skill training), reflecting activity (or experience) related plasticity [51, 53–55]. Based on these findings from motor skill training literature, it is plausible that EMST, which involves a degree of skill acquisition and neural adaptation, may produce similar neuroprotective benefits. It is important to note that the neuroprotective effects of EMST have been discussed [10], and the prospects remain appealing yet speculative.

In addition to the direct sensorimotor benefits, EMST may also promote integration between cognitive and sensorimotor systems. The observed involvement of regions such as the middle frontal gyrus, right insula, anterior cingulate, and thalamus, areas linked to cognitive, sensory, and multimodal processing, suggests that enhanced cognitive-sensorimotor integration may represent a third mechanism contributing to the observed improvements in swallowing function. Furthermore, emerging evidence highlights the roles of cognitive and attention-driven processing in swallowing sensorimotor control [56]. The activation in these areas suggests that cognitive factors, such as attention and executive control, may support the efficient coordination of the sensorimotor processes required for swallowing and potentially other upper aerodigestive functions. We speculate that improved cognitive-sensorimotor integration due to EMST may enhance the brain's feedback and feedforward loops, resulting in adaptive and precise motor execution for tasks, such as swallowing.

Increasing evidence suggests that resistance training not only enhances physical strength but also improves cognitive function [57–59]. These cognitive improvements are thought to stem from the neuroplastic adaptations that occur in response to exercise training, including the upregulation of BDNF and other growth factors [60–64]. Our previous research has demonstrated an increase in circulating serum BDNF and IGF-1 levels following EMST [9], along with a strong positive correlation between circulating BDNF levels and expiratory muscle strength gains [18]. Given these findings and current evidence showing increased activation in brain regions associated with cognitive and executive functions, we speculate that prolonged EMST may lead to cognitive improvements similar to those observed with other forms of resistance training. While drawing conclusions without behavioral evidence would be premature, longitudinal studies investigating the cognitive benefits of EMST may uncover additional effects beyond improvements in expiratory muscle strength and upper aerodigestive function.

## 5. Conclusion and Future Directions

Our study provides preliminary evidence that EMST can modulate neural networks associated with swallowing, potentially improving a range of upper aerodigestive functions through three top–down mechanisms: enhanced sensorimotor excitability and cortical representation, motor learning, and improved cognitive-sensorimotor integration. However, given the preliminary nature of our findings, we acknowledge our study's limitations related to a smaller sample size. We also recognize the necessity of including a more diverse participant pool. Future experimental studies will include a control group (or a sham training group) to increase the generalizability of our findings. Moreover, prospective randomized controlled trials are needed to establish causal relationships.

In addition to strengthening expiratory muscles and promoting neuroplastic changes, EMST may also influence specific sensory functions. EMST training has been linked to changes in sensory perception, such as urge-to-cough in clinical populations [32, 65], which is critical for airway protection in dysphagia. While our studies did not directly measure airway sensitivity or afferent feedback, it is plausible that these changes in cough sensitivity are related to EMST-induced remodeling of central and peripheral components of mechanosensory pathways. The repetitive, effortful nature of EMST could lead to altered sensitivity or responsiveness of airway mechanoreceptors, such as those in the upper aerodigestive system. Such adaptations may improve sensory gating or sensorimotor integration, critical for reflexive and volitional airway protective behaviors (such as cough). Future studies could more directly assess changes in airway sensory thresholds or afferent signaling (via laryngeal sensory testing or evoked potentials) to further elucidate this sensory dimension of EMST.

The strong activation of brain regions linked to motor learning processes provides preliminary evidence indicating that EMST involves dynamic learning and may offer neuroprotective benefits over time, potentially improving both motor performance and overall brain health. However, while these initial findings are encouraging, further investigation is necessary, and we recommend that future studies assess the gray matter and white matter volumetric changes resulting from EMST to support these claims. Furthermore, we recommend investigating the effect of EMST on intrinsic brain activity through resting state functional connectivity and its potential cognitive benefits. In the present study, post-training fMRI scans were acquired within 3 days of completing the 4-week EMST protocol, allowing us to capture immediate (short-term) neuroplastic changes. However, we did not include follow-up or detraining scans, so it remains unknown whether the observed increases in activation across brain regions persist over time without continued training. Based on existing literature in motor learning and neurorehabilitation, it is likely that ongoing or maintenance EMST would be necessary to sustain both the functional and neuroplastic gains. Studies incorporating long-term follow-ups are needed. It would be interesting to extend this work to healthy aging individuals, clinical populations, including individuals with neurodegenerative diseases, such as Parkinson's disease, stroke survivors, and pediatric populations with developmental motor impairments. Such studies programmatically could answer key questions about the long-term impact of EMST on functional improvements, brain health, and the mechanisms through which it exerts neuroplastic and neuroprotective effects.

## Figures and Tables

**Table 1:** Coordinates, cluster size, and effect descriptors for regions associated with the effect of EMST on swallow task.

Sl No.	Cluster	Coordinates (MNI152-2009)			Volume (mm^3^)	Direction of change	Cluster-wise *p*-value
		RL	AP	IS			
1	Left primary and secondary visual areas	−18	68	−11	598	PT > BL	0.003
2	Right middle frontal gyrus	−32	−58	16	193	PT > BL	0.014
3	Left postcentral gyrus	62	26	29	164	PT > BL	0.007
4	Right cerebellum (VI, Crus I, II, VIII, IX)	−21	83	−27	132	PT > BL	0.003
5	Left middle frontal gyrus	18	−53	16	74	PT > BL	<0.001
6	Right insula	−48	−1	−3	55	PT > BL	0.017
7	Left SMA	10	−2	78	49	PT > BL	0.005
8	Left precentral gyrus	47	14	28	42	PT > BL	0.003
9	Right SMA	−1	−1	73	28	PT > BL	<0.001
10	Right thalamus	−3	−7	9	23	PT > BL	0.006
11	Right anterior cingulate	−3	−10	26	21	PT > BL	0.04
12	Left cerebellum (dorsal dentate nuclei)	10	45	27	18	PT > BL	<0.001

*Note:* The reported *p* values reflect the mean of voxel-wise *p* values within each significant cluster.

Abbreviations: AP, anterior–posterior; BL, baseline; IS, inferior–superior; PT, post-training; RL, right–left.

## Data Availability

The data that support the findings of this study are available from the corresponding author upon reasonable request.
